# Screening of Cervical Cancer with Self-Collected Cervical Samples and Next-Generation Sequencing

**DOI:** 10.1155/2018/4826547

**Published:** 2018-11-14

**Authors:** Yubo Fan, Yifan Meng, Shuo Yang, Ling Wang, Wenhua Zhi, Cordelle Lazare, Canhui Cao, Peng Wu

**Affiliations:** Department of Gynecologic Oncology, Tongji Hospital, Tongji Medical College, Huazhong University of Science and Technology, Wuhan, Hubei, China

## Abstract

Cervical cancer is the second leading cause of death in female genital malignancies. Persistent infection with high-risk HPV is closely related to cervical intraepithelial neoplasia (CIN). Wide-scale HPV screening has already been implemented in developed countries. However, with advances in HPV testing methods, there are presently no better methods for the management of the increasing number of high-risk HPV-positive women except for periodic review. In order to improve screening coverage and achieve better triage of those women, we present current HPV testing methods with self-collected cervical samples, focusing on recent advances in next-generation sequencing (NGS) technologies as a promising screening technology for cervical cancer precursors.

## 1. Introduction

Most of HPV infections are transient, while few persist and eventually induce carcinogenesis [1]. In developed countries, cytology combined with HPV testing is the primary screening method for cervical cancer. However, in low-resource areas with a high incident rate of cervical cancer, lack of infrastructure limits the participation in screening programs. Many countries are struggling with nonorganized cervical cancer screening programs with very low coverage of the targeted screening population [2]. Taking these barriers into consideration, self-collected sampling has been shown to facilitate access to cervical screening without extensive infrastructure and is suitable for HPV testing, which could enable good coverage and achieve good attendance. Due to the high sensitivity of cervical cancer precursors, primary high-risk HPV screening alone was recommended as an alternative to the current screening method in 2015. This alternative may lead to early detection and improve the quality of patients' life. Unfortunately, HPV testing has a noticeable false-positive rate, which leads to repeated colposcopy, thus increasing the mental and economic burden of patients. Hence, most guidelines do not recommend further intervention for women with persistent high-risk HPV infection, but excessive and frequent screening and relevant treatments are very common [3]. Therefore, it becomes urgent to achieve better triage for primary HPV infections. The emergence of next-generation sequencing (NGS) technologies provides an opportunity to directly examine viral diversity in clinical samples without previous sequence information [4]. It has been progressively applied to HPV typing and has proven to be highly accurate and reproducible with high sensitivity to detect and identify multiple HPV-type infections [5]. Here, we describe HPV-based screening methods and NGS technologies for the early detection of CIN.

## 2. Cytology and HPV Testing

To date, cytology and HPV testing were the most common methods for cervical cancer screening in clinical practice. Due to cytology's high sensitivity for detection of cervical cancer precursors, it is now used for triage of HPV-positive women to avoid unnecessary referral to colposcopy. However, cytology's subjectivity and missed diagnosis add to the false-negative rate because it relies on artificial diagnosis. Since high-risk HPV is an adequate etiologic agent for cervical lesions, high-risk HPV DNA genotyping could identify those high-risk HPV-positive women who are likely to have CIN. This has been successfully applied to cervical cancer screening programs. Based on the high sensitivity and the negative predictive value, it allows for better management of HPV-negative women who are unlikely to develop cervical cancer over the next 5–10 years. The first high-risk HPV DNA testing approved by the US FDA was Hybrid Capture II (HC2), used for HPV genotyping. Other assays such as Cobas®, based on RT-PCR, have been approved by FDA for cervical cancer screening of women aged 30 years and above combined with cytology [6]. Despite this, specificity is low, along with the absence of HPV genotyping in many CIN3+ cases. Meanwhile, it cannot differentiate between a transient and persistent infection. 90% of HPV-infected women are able to spontaneously clear infections. Moreover, the lack of cytological abnormalities in most of them increases unnecessary colposcopy referral and psychological distress of those patients. When combined with HPV testing, sensitivity reaches up to 90% while 5-year risk for precancerous cervical lesions is nearly negligible.

## 3. p16/Ki67 Dual Staining

Most CIN1 showed p16-positive staining, but squamous metaplasia cervical cells under normal physiological conditions were occasionally also positive. Ki67 is a proliferation marker which confers additional specificity for CIN. Therefore, dual staining of p16 and Ki67 helps to identify truly malignant cells. Compared with HPV testing or single p16 staining, the sensitivity of dual staining in the detection of CIN2 and greater is significantly enhanced, while maintaining the same specificity [7]. HPV+/p16+ women were at a high risk for CIN3+ after 3 years of persistent infection [8]. Data from a large Italian screening trial suggested immediate colposcopy referrals to HPV16/18+ women combined with dual-stained positive p16 and Ki67 tests. This may reduce the false-positive rate of HPV testing, which allows better triage for HPV-positive women.

## 4. Other Markers

Persistent infection may lead to the integration of the HPV genome into the host chromosome, causing the termination of normal viral life cycle and overexpression of E6 and E7 oncoproteins, by methylation of CpG sites. HPV integration often occurs in the early stage of CIN. Now that HPV DNA testing is only fit for primary screening, the screening of molecular abnormalities or biomarkers is now in the ascendant. RT-PCR-based E6/E7 mRNA testing not only provides quantitation of viral load but also indicates its transcriptional activity, meaning that E6/E7 mRNA testing holds a prognostic value. It is a biomarker of significant dysplasia and cervical cancer [9]. Genome-wide studies of high-risk HPV have demonstrated that methylations of viral CpG sites may represent the key of transformation from HPV transient infection to cervical cancer precursors [10]. More than 20 genes, including CADM1/MAL, PAX1, SOX1, ZNF582, PCDHA4, and PCDHA13, have been confirmed to be associated with the lesions of CIN2 and greater. High accuracy and specificity made it promising for the early diagnosis of cervical cancer. But first the study should be extended to methylation markers of other types of high-risk HPV associated with cervical cancer as well as detection of their sensitivity and specificity in a large-scale clinical trial. Now, artificial intelligence is used in order to weigh the importance of different HPV genotypes in predicting cervical dysplasia persistence/recurrence, like the artificial neuronal network (ANN) analysis [11].

## 5. Self-Collected Sampling

In low-resource areas without extensive infrastructure for cytopathological assessment, HPV testing can be done on a vaginal sample taken by the women themselves. This may offer opportunities to reach those who are reluctant to undergo gynecological examinations. Compared with samples acquired in the clinical setting, the sensitivity of HPV testing on self-samples is lower and less specific for the exclusion of CIN2 or worse [12]. On the other hand, self-collected specimens can be used for HPV-based screening, providing sensitivity and specificity comparable with those of clinician-collected specimens and detecting disease earlier than cytology. When positive cytology on clinician-collected samples is defined as LSIL or worse, HPV testing on self-samples is more sensitive in the detection of CIN2 or worse and CIN3 or worse [13].

Thus, for HPV testing based on DNA or RNA, clinician-taken samples rather than self-samples should be chosen because of superior sensitivity and specificity. While in a cytology-based or HPV-based screening program, HPV testing on a self-sample can be suggested as an additional strategy to reach women not participating in the regular screening program. Since self-collected sampling can economize cost compared with clinician-collected sampling, it could achieve better coverage in low-resource areas. However, women whose self-collected specimens test positive for high-risk HPV require additional triage testing because the specificity of assays for high-risk HPV is insufficient to justify direct referral for colposcopy in all cases [2]. The self-collected HPV test has only been evaluated cross-sectionally. Longitudinal evaluations of its ability are still needed to capture disease missed by colposcopy.

## 6. Next-Generation Sequencing (NGS)

The emerging NGS technology is a promising method for the characterization of HPV genotypes, providing a deeper understanding for mechanisms of carcinogenicity. NGS is a massively parallel high-throughput methodology, including whole genome sequencing (WGS), whole exome sequencing (WES), RNA sequencing (RNA-seq), miRNA sequencing (miRNA-seq), and whole genome bisulfite sequencing (WGBS), which has broad application prospects. It could detect viral existence and a wide range of HPV genotypes from poor clinical samples, as well as uncharacterized HPV types, thus being more specific than hybridization-based methods and breaking through the limitation of minor HPV types. Compared with PCR-based assays, NGS shows high sensitivity and is suitable for detecting multiple HPV infections [14]. In fact, multiple infections are common, and intergenotypic competition between them or more effective immune responses may reduce high-risk cervical cancer rates [15]. Thus, NGS could probably achieve better triage for high-risk HPV-positive women.

Integration may be the key step from transient to persistent infection. Since the integration rate is up to 76.3% of cervical cancer cases and positively correlated with CIN grades [16], it can be used as a specific biomarker for early diagnosis. With the development of NGS, genome-wide profiling of HPV integration sites is becoming feasible. Integration occurs in regions of microhomology among the HPVs and host genome. An innovative NGS assay can detect viral-cell junctions by capturing all viral-containing molecules after hybridization with customized HPV probes and deep sequencing [17]. It can also provide single-molecule CpG methylation levels to help unravel the physiological role of methylation in cervical cancer development. Further research on how changes in the viral and host methylome are associated with cervical cancer development should provide mechanistic insights facilitating prevention and treatment [18]. As an alternative technique for carcinogenic HPV detection, NGS rapidly developed new techniques like high-throughput viral integration detection (HIVID) and TEN16 methodology breaking the limitation of small sample sizes. There is a bright prospect to apply them to large-scale HPV screening and better triage of high-risk HPV-positive women and then avoid unnecessary referral to colposcopy.

However, there are two main issues which should be considered. Firstly, the increased identification of integration loci is dependent on the development of novel detection technologies such as HIVID. Nevertheless, the consistency and repeatability of this technology should be validated, since the HIVID technology stems from WGS and some integration sites could not be confirmed by WGS. Secondly, the practicality and applicability of NGS should be evaluated. Over the years, the high cost associated with NGS has hindered its clinical application. However, the cost has dramatically decreased in recent years and is expected to continue to decrease in the near future. With the aid of artificial intelligence, automation, and standardization, NGS cost and processing time will eventually reach levels practical for its application in HPV screening.

## 7. Conclusion

HPV testing methods could detect infections which link to cervical cancer at an earlier stage, making it possible for the early treatment of HPV-positive women to improve prognosis and reduce mortality. The similar performance of the self-collected and the clinician-collected HPV test for the detection of prevalent and incident disease indicates that this could represent an invaluable tool for improving screening coverage, especially in low-income and middle-income countries with substantial burden of cervical cancer. In the future, self-samples might reach similar accuracy as clinician-collected samples in HPV tests. NGS is cost-effective in the early diagnosis of CIN. It can be used for (i) precise HPV typing, particularly suitable for multiple infection and large numbers of samples, (ii) detecting biomarkers with high sensitivity and specificity, (iii) interpreting mechanisms of carcinogenesis, and (iv) facilitating new therapy development. Ongoing large prospective clinical studies are testing the sensitivity, specificity, and positive and negative predictive values of NGS. However, HPV screening is facing great problems like how to manage HPV-positive triage-negative women and overtreatment. No assay alone could determine the risk of cervical cancer, so triages are still indispensable. Despite these, with all the aforementioned methods, early diagnosis and treatment will definitely improve the prognosis of cervical cancer in the bright future.
